# Inhibition of Angiogenesis *In Vitro* by Chebulagic Acid: A COX-LOX Dual Inhibitor

**DOI:** 10.1155/2013/843897

**Published:** 2013-10-31

**Authors:** A. P. Athira, A. Helen, K. Saja, P. Reddanna, P. R. Sudhakaran

**Affiliations:** ^1^Department of Biochemistry, University of Kerala, Thiruvananthapuram, Kerala 695581, India; ^2^National Institute of Animal Biotechnology, University of Hyderabad Campus, Hyderabad 500046, India; ^3^Department of Animal Sciences, University of Hyderabad, Hyderabad 500046, India; ^4^Department of Computational Biology and Bioinformatics, State Inter-University Centre for Excellence in Bioinformatics, University of Kerala, Thiruvananthapuram, Kerala 695581, India

## Abstract

Angiogenesis is a crucial step in the growth of cancer and its metastasis. It is regulated by several endogenous factors which may stimulate or inhibit the new blood vessel growth. Besides these endogenous factors, several exogenous factors including some natural compounds are known to modulate angiogenesis. Angiogenesis being a potential target for drugs against a number of pathological conditions, search for compounds from natural sources that can affect angiogenesis is of great interest. The objective of our present study was to understand the effect of chebulagic acid, a COX-LOX dual inhibitor isolated from the fruits of *Terminalia chebula* Retz., on angiogenesis. The model systems used were rat aortic rings and human umbilical vein endothelial cells. The results showed that chebulagic acid exerts an antiangiogenic effect. This was evidenced from decreased sprouting in rat aortic rings and decrease in biochemical markers in endothelial cells treated with chebulagic acid. It downregulated the production of CD31, E-selectin, and vascular endothelial growth factor in human umbilical vein endothelial cells in culture (HUVEC). Further studies to understand the molecular mechanism of action of chebulagic acid revealed that CA exerts its anti angiogenic effect by modulating VE cadherin-*β* catenin signalling in human umbilical vein endothelial cells.

## 1. Introduction

Angiogenesis, the regulated formation of new blood vessels from preexisting ones, plays a crucial role in organogenesis, advanced embryonic development, wound healing, and growth and action of female reproductive organs. Although angiogenesis is essential during these processes, in adulthood, it has a limited role in normal physiology and is mostly linked to pathological conditions such as tumorigenesis, rheumatoid arthritis, obesity, and diabetic retinopathy [1].

Angiogenesis is a complex and orderly process that involves cell-cell and cell-extracellular matrix interactions, which is controlled by a balance between angiogenic and angiostatic factors. Disruption of this balance leads to aberrant angiogenesis resulting in pathological conditions, arising due to hypo- or hyperangiogenesis [2]. The activation of endothelial cells, the initial step of angiogenesis, occurs when positive regulators predominate. The endothelial quiescence is achieved and maintained by the dominance of negative regulators [3, 4].

Apart from these endogenous factors, several exogenous factors are known to modulate angiogenesis. Naturally occurring bioactive compounds are gaining attention as therapeutic agents since they modulate biological processes that underlie many disease states. Angiogenesis being a potential target for drugs against a number of pathological conditions, search for compounds from natural sources that can affect angiogenesis is of great interest. Much of the recent research is focused on therapeutic approach to identify natural compounds which are able to modulate angiogenesis. Inhibition of angiogenesis is now considered to be one of the most promising strategies leading to the development of new antineoplastic therapies. 

Chebulagic acid (CA), a benzopyran tannin present as one of the major constituents in the fruits of *Terminalia chebula *Retz., is our compound of interest. The fruit powder of *Terminalia chebula *Retz. (TC) is used in India to treat several diseases ranging from digestive, coronary disorders to allergic and infectious diseases like cough and skin disorders. Recent reports showed that CA exerts a potent anti-inflammatory effect on LPS stimulated macrophages by inhibiting NF*κ*B activation and MAPK phosphorylation [5] and is also a potent COX-2 and 5-LOX dual inhibitor [6]. Since angiogenesis and inflammation are codependent, the main objective of our study was to examine the effect of chebulagic acid on angiogenesis. The influence of chebulagic acid on angiogenesis was studied in detail using *in vitro* model systems, and the results presented here show its antiangiogenic effect.

## 2. Materials and Methods

### 2.1. Materials

Collagenase, MCDB 131 medium, minimum essential medium, antibiotic-antimycotic solution, o-phenylenediamine dihydrochloride, diaminobenzidine, Tris, glycine, protease inhibitor cocktail, bovine serum albumin (BSA), fetal bovine serum, monoclonal antibodies against CD31, E-selectin, VEGF, MMP 2, MMP 9, VE cadherin, *β* catenin, and horseradish-peroxidase-(HRP-) conjugated secondary anti-mouse and anti-rabbit antibody were purchased from M/S Sigma-Aldrich Chemicals (St. Louis, MO, USA). Nitrocellulose membranes were obtained from Bio-Rad Laboratories (Hercules, CA, USA). Tissue culture plastic wares were purchased from NUNC A/S (Roskilde, Denmark).

### 2.2. Methods

#### 2.2.1. Isolation of Chebulagic Acid by RP-HPLC from Ethanolic Extract of TC Fruits

Chebulagic acid was extracted and purified from the dried fruits of *Terminalia chebula *as described earlier [6]. Briefly, the finely powdered dried fruits were extracted with absolute alcohol for an hour and centrifuged at 5000 ×g for 5 minutes. The lyophilized ethanolic extract was redissolved at 20 mg/mL in absolute alcohol and subjected to RP-HPLC by employing C18 column (shim-pack column 250 × 4.6 mm and particle size 5 *µ*m) with 1 mL/min flow rate, and the eluants were monitored at 280 nm. The mobile phase consisted of a complex gradient of solvent A (water : acetic acid (1000 : 1)) and solvent B (acetonitrile : acetic acid (1000 : 1)). All the fractions were collected and CA was identified by LC-MS, IR, and NMR spectra. The purity of the CA obtained was >98.0%. Stock solution was prepared in DMSO.

#### 2.2.2. Aortic Ring Assay

Aortic ring assay was carried out as described previously [7]. Rat thoracic aortas 1 mm thick were maintained in minimum essential medium containing 10% FBS at 37°C in a 95% air and 5% CO_2_ atmosphere in a Sanyo CO_2_ incubator. Thoracic aortas were taken from 4- to 6-week-old Sprague-Dawley rats and immediately transferred to a sterile tube containing minimum essential medium. After removing the fibroadipose tissue around the aorta, it was sectioned into approximately 1 mm long aortic rings with a sharp sterile surgical blade. The rings were placed in culture plates supplied with MEM containing FBS with or without CA. Six to eight aortic rings were used in each set and the sprouting of cells from the aortic rings was examined microphotographically using a Leica microscope. The extent of sprouting was quantitated using QWIN Leica software. All experiments were carried out in accordance with the guidelines of the Institutional Animal Ethics Committee.

#### 2.2.3. Isolation and Culture of Human Umbilical Vein Endothelial Cells

Endothelial cells were isolated by collagenase perfusion of umbilical vein as described before [8, 9]. The cells were maintained in culture at 37°C in a 95% air and 5% CO_2_ atmosphere in a Sanyo carbon dioxide incubator in MCDB 131 medium containing 10% FBS. Stock solutions of CA were diluted with PBS and supplemented to medium to study their effect on cells in culture.

#### 2.2.4. Enzyme-Linked Immunosorbent Assay (ELISA)

Indirect ELISA was carried out using specific primary antibody (1 : 1000 dilution) and HRP-conjugated secondary antibody (1 : 1000 dilution) [10]. The amounts of CD31, VE cadherin, and *β* catenin in cell lysate and VEGF, E-selectin, MMP 9, and MMP 2 in medium were quantitated by ELISA. Cell extract and cell culture medium precoated onto ELISA plates served as antigen. o-Phenylenediamine dihydrochloride was used as the substrate. The concentrations of these antigens were estimated by measuring the absorbance of the coloured HRP product spectrophotometrically at 490 nm in a multiwell microplate reader (Thermo Scientific Multiskan Spectrum). 

#### 2.2.5. Western Blot

The production of various proteins was determined by western blot analysis [11]. Proteins were separated in a 10% polyacrylamide gel, transferred onto nitrocellulose membranes, and probed using specific monoclonal anti-human antibody (dilution 1 : 1000) followed by secondary anti-mouse IgG conjugated to HRP (dilution of 1 : 1000). The bands were detected by staining with 3,3′-diaminobenzidine, and the relative intensity of bands was quantitated using Bio-Rad Quantity One version 4.5 software in a Bio-Rad gel doc.

#### 2.2.6. Distribution of *β* Catenin

Cell pellets were resuspended in 500 *μ*L PBS ice-cold nuclear buffer (150 mM NaCl, 150 mM sucrose, 20 mM HEPES, pH 7.4, 5 mM KCl, 2 mM dithiothreitol, 1 mM MgCl_2_, 0.5 mM CaCl_2_, 0.1 mM PMSF, and protease inhibitors) [12]. The suspension was gently mixed with a pipette on ice for 3 min and then centrifuged at 500 g for 10 min to pellet the nuclei. The cytosolic supernatant was centrifuged for 60 min at 13000 g at 4°C. Protein equivalent amount of cytosolic and nuclear fractions was used for ELISA.

#### 2.2.7. Statistical Analysis

All the data were expressed as mean with standard error of mean. The statistical significance of difference was measured by Newman-Keuls multiple comparison one-way ANOVA and *t*-test using Graphpad Prism software. A value of *P* < 0.05 was considered significant.

## 3. Results

### 3.1. Effect of Chebulagic Acid on Angiogenesis: Aortic Ring Assay

**Figure 1:**
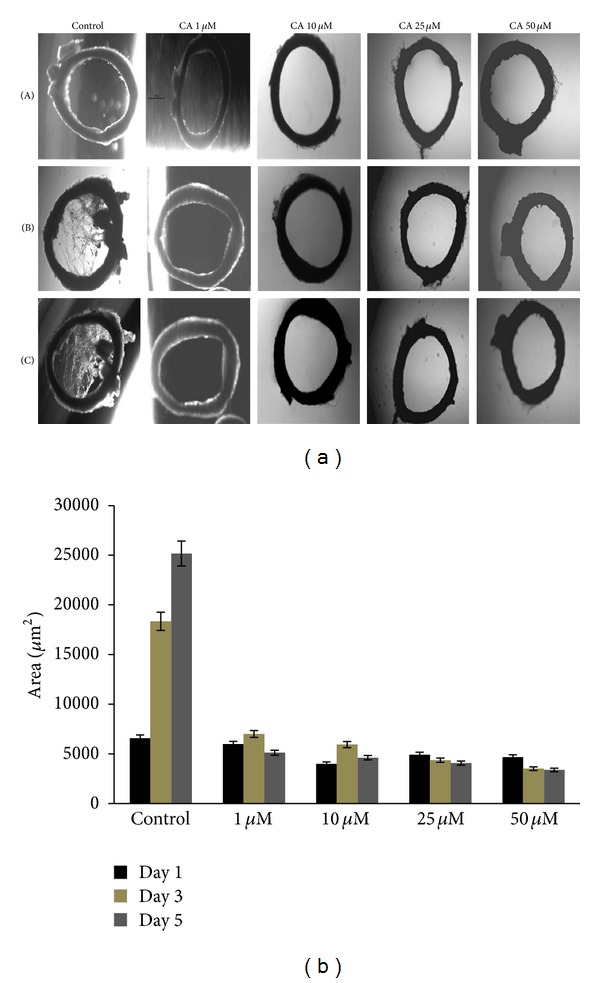
(a) Effect of chebulagic acid on sprouting of rat aortic rings. Rat aortic ring explants were maintained in culture in the presence of 10% fetal bovine serum (FBS). Different concentrations of chebulagic acid (1 *μ*M, 10 *μ*M, 25 *μ*M, and 50 *μ*M) were supplemented to the medium and was monitored sprouting, under a microscope at different time intervals. The morphological changes were visualized and photographed under a microscope (×4) on the 1st (A), 3rd (B), and 5th (C) days. Each set was done in replicates, and the microphotographs from a representative experiment are given. (b) The sprouts were quantitated using QWIN software (Leica) and expressed in area in *µ*m^2^. Values given are the average of five experiments ± SEM, statistically significant compared to control (*P* < 0.05).

The effect of chebulagic acid on endothelial sprouting was studied by maintaining rat aortic rings in MEM supplemented with 10% FBS and chebulagic acid at various concentrations. Endothelial sprouting was not observed in aortic rings treated with chebulagic acid. In control aortic rings, endothelial sprouting was observed at 72 hours of culture and the sprouting increased significantly with the progression of culture, while in aortic rings treated with chebulagic acid at concentration as low as 1 *μ*M, sprouting was not observed at 72 hours and no significant sprouting was observed with the progression of culture up to the fifth day, indicating an inhibitory effect of chebulagic acid (Figures 1(a) and 1(b)). 

### 3.2. Effect of Chebulagic Acid on Endothelial Cells in Culture

**Figure 2:**
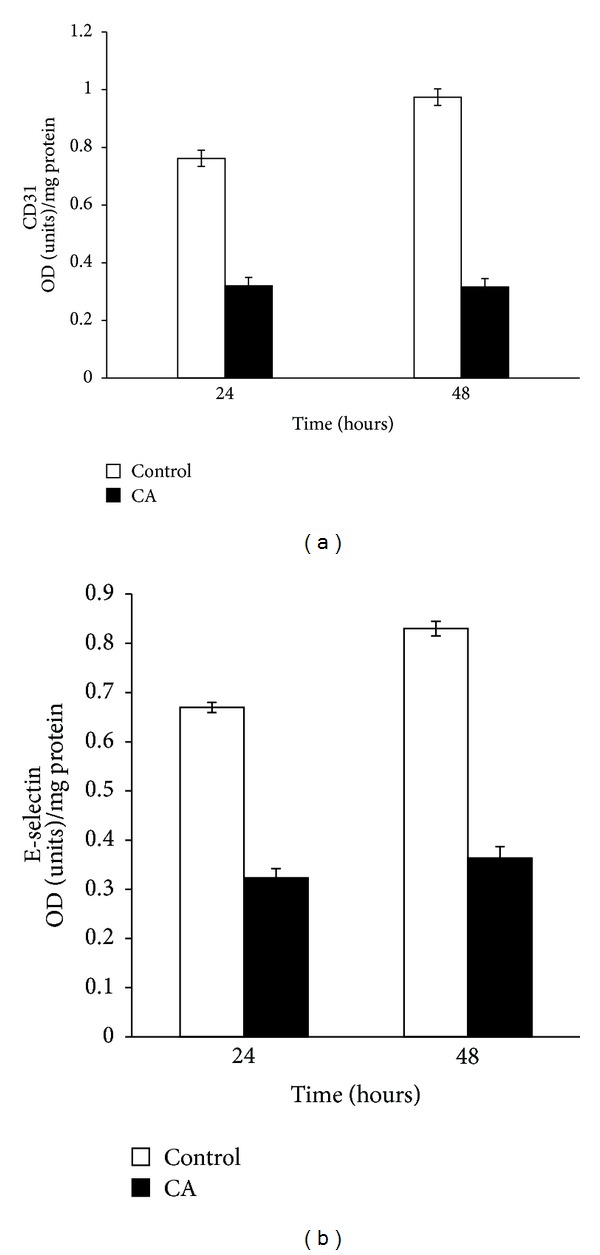
Effect of CA on the expression of CD31 and E-selectin. HUVECs were maintained in culture in MCDB 131 medium containing 10% FBS supplemented with 25 *μ*M CA for 48 hours. The levels of cell-associated CD31 (a) and E-selectin secreted into the medium (b) at 24 and 48 hours were estimated by ELISA using anti-CD31 (1 : 1000) and anti-E-selectin (1 : 1000) antibody, respectively. Values given are the average of duplicate analysis of 5 experiments ± SEM (*P* < 0.05).

In order to further examine the effect of chebulagic acid, HUVECs in culture were treated with CA in the presence of serum. Cells did not show angiogenic phenotype in treatment with chebulagic acid even at 72 hours in culture, while control cells developed cell-to-cell contact at 48 hours of culture itself. The production of endothelial markers CD31 and E-selectin (Figure 2) was also analyzed. The levels of these markers were found to be decreased in HUVECs treated with CA in the presence of serum compared to control cells. Nearly 30% reduction in the levels of E-selectin was caused by CA at concentration as low as 1 *μ*M; with increase in its concentration, there was progressive inhibition, and about 70% inhibition was produced by 25 *μ*M of CA. Hence, for further studies on the effect of CA on HUVECS in culture, 25 *μ*M of CA was used. A significant reduction in the levels of CD31 was also observed in cells treated with CA. This further suggests that chebulagic acid in the presence of serum exerted an antiangiogenic effect. 

### 3.3. Effect of Chebulagic Acid on the Production of Angiogenic Factor VEGF

**Figure 3:**
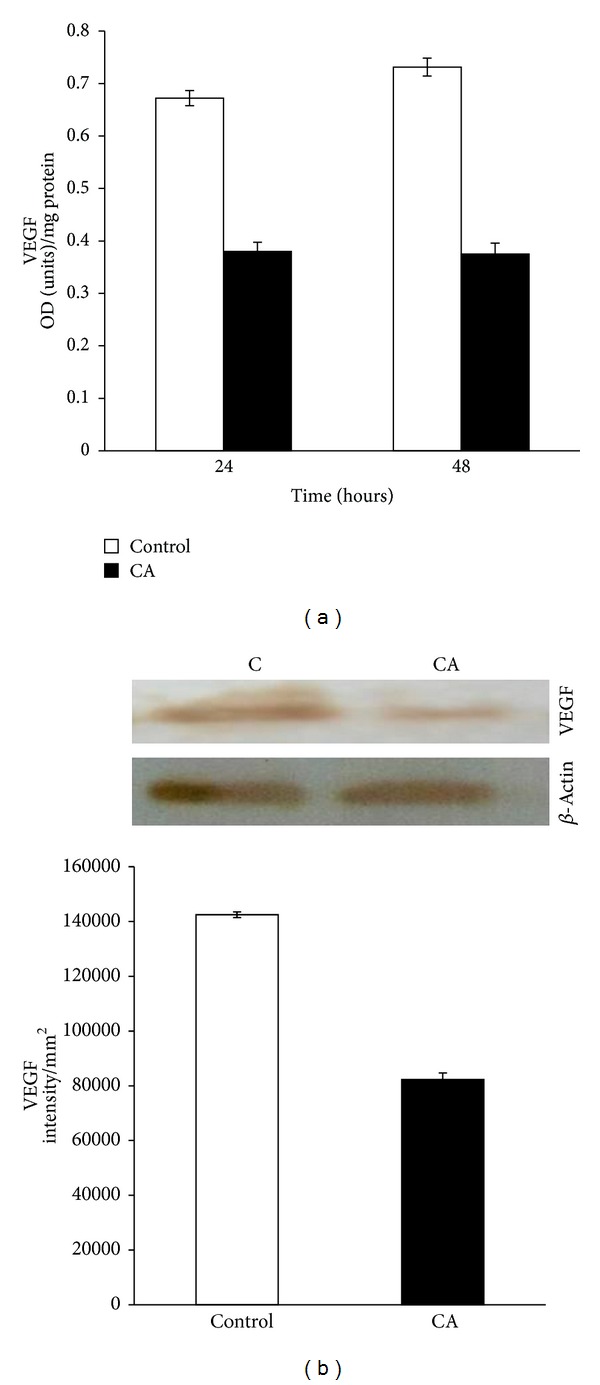
Effect of CA on the expression of VEGF. HUVECs were maintained in culture in MCDB 131 medium containing 10% FBS supplemented with 25 *μ*M CA for 48 hours. (a) The level of VEGF secreted into the medium was estimated using anti-VEGF antibody. Values given are the average of duplicate analysis of 5 experiments ± SEM (*P* < 0.05). (b) Western blot to analyze the production of VEGF by control cells (C) and chebulagic acid treated cells (CA) at 48 hours. The intensity of the immunoblotted bands was measured, normalised to intensity of internal control actin, and expressed in intensity units/mm^2^.

The molecular mechanism of the effect of chebulagic acid on angiogenesis was further analyzed by studying the production of VEGF, which is an important endothelial cell-specific angiogenic factor. Both ELISA and western blot showed that VEGF production was significantly decreased in cells treated with chebulagic acid compared with control cells (Figure 3). There was steady decrease in the levels of VEGF secreted into the medium by cells treated with increasing concentration of CA; about 30% inhibition was produced by CA at a concentration of 1 *μ*M.

### 3.4. Effect of Chebulagic Acid on the Production of Matrix Metalloproteinases

**Figure 4:**
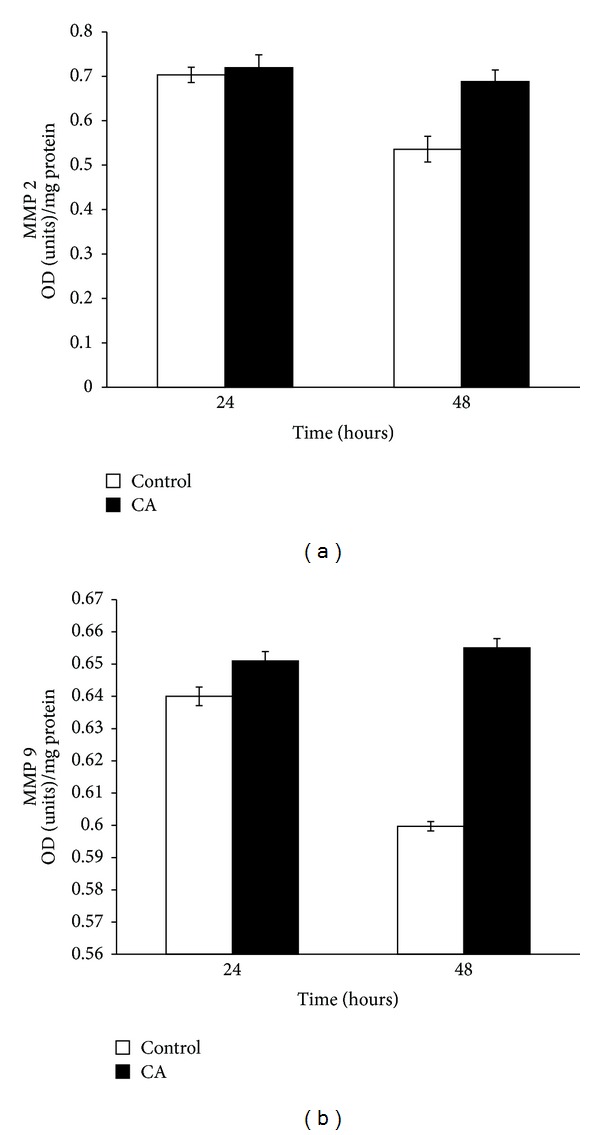
HUVECs maintained in culture in MCDB 131 containing 10% FBS for 48 hours were treated with 25 *μ*M CA. The level of MMP 2 and MMP 9 in the medium was estimated by ELISA using anti-MMP 2 (1 : 1000) (a) and MMP 9 (1 : 1000) (b) antibody, respectively. Values given are the average of duplicate analysis of 5 experiments ± SEM (*P* < 0.05).

In order to examine whether there is any association between MMP production and angiogenesis, medium from HUVECs treated with chebulagic acid and their controls was subjected to ELISA. There was a downregulation in the levels of MMP 2 and MMP 9 in controls with progression of culture, while the amount of these enzymes remained high throughout the culture in cells treated with chebulagic acid. The activity of MMP 2 on each day was significantly high in cells treated with chebulagic acid when compared to respective control (Figure 4).

### 3.5. Effect of Chebulagic Acid on the Production of VE Cadherin

**Figure 5:**
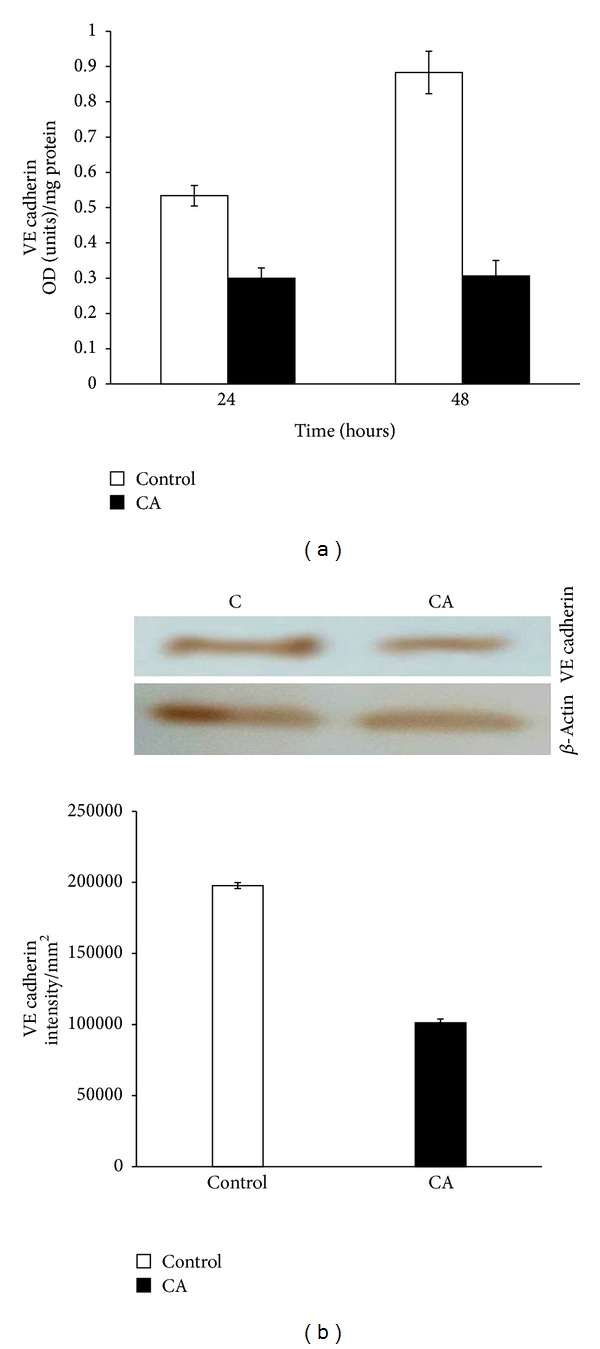
Effect of CA on the expression of VE cadherin. HUVECs were maintained in culture in MCDB 131 medium containing 10% FBS supplemented with 25 *μ*M CA for 48 hours. (a) The level of VE cadherin in the cell layer was estimated using anti-VE cadherin antibody. Values given are the average of duplicate analysis of 5 experiments ± SEM (*P* < 0.05). (b) Western blot to analyze the production of VE cadherin by control cells (C) and chebulagic acid treated cells (CA) at 48 hours. The intensity of the immunoblotted bands was measured, normalised to that of internal control, and expressed in intensity units/mm^2^. Values given are mean of 5 experiments ± SEM.

In order to understand whether chebulagic acid modulates the production of VE cadherin, the intercellular cell adhesion molecule involved in endothelial cell-cell interaction and angiogenesis, control and chebulagic acid treated cells were subjected to ELISA and western blot. The results showed that there was a significant decrease in the production of VE cadherin in cells treated with chebulagic acid compared to control (Figure 5). 

### 3.6. Effect of Chebulagic Acid on the Distribution of *β* Catenin

**Figure 6:**
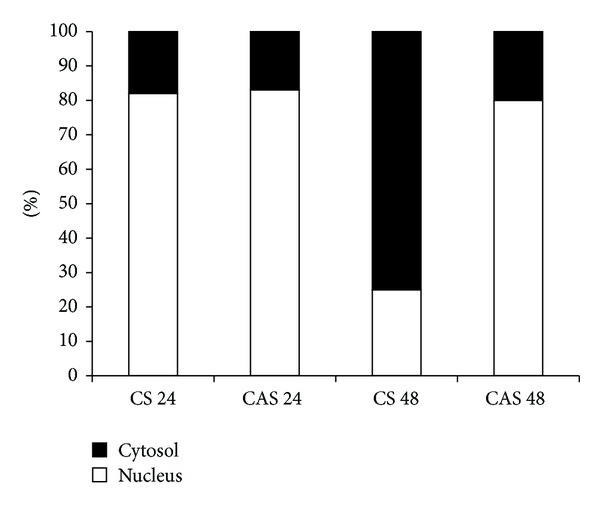
Effect of CA on the distribution of *β* catenin. HUVECs maintained in culture in MCDB 131 medium containing 10% FBS were supplemented with 25 *μ*M CA. The level of *β* catenin in the nucleus and cytosol at 24 and 48 hours was estimated by ELISA using anti-*β* catenin antibody (1 : 1000) and expressed as percent of total. CS 24 and CS 48: control at 24 and 48 hours. CAS 24 and CAS 48: chebulagic acid at 24 and 48 hours.

*β* catenin is one of the accessory proteins associated with the cytoplasmic tail of VE cadherin aiding the stabilization of cell-cell adhesion. Analysis of the relative levels of *β* catenin in the nucleus and cytoplasm of the cells treated with chebulagic acid showed a higher level of *β* catenin in the nucleus during the initial and later stages of culture, whereas in control cells *β* catenin level in cytosol increased and that in nucleus decreased correspondingly during the later stages of culture (Figure 6).

## 4. Discussion

Recent studies have indicated the major role of cyclooxygenase, COX-2, in neoplastic transformation and cancer growth, by downregulating apoptosis and promoting angiogenesis, invasion, and metastasis, and, therefore, selective inhibitors, coxibs, have a potential role in the prevention and treatment of cancer [13]. However some of these inhibitors cause side effects limiting their therapeutic use. Searching for new compounds targeting eicosanoid pathways critical in inflammatory disorders led to the identification of compounds that inhibit the key enzymes of these pathways. Chebulagic acid is a COX-LOX dual inhibitor, that showed antiproliferative activity against different cancer cell lines and induced apoptosis of COLO-205 cells [6]. The results presented here indicate that chebulagic acid exerts an antiangiogenic effect in rat aortic rings and endothelial cells in serum supplemented condition. This was evidenced by decreased cell sprouting in rat aortic rings treated with chebulagic acid compared to control. The antiangiogenic effect of chebulagic acid was further studied by examining the production of endothelial specific markers like CD31 and E-selectin. CD31 is an endothelial marker that has been shown to increase in endothelial cells undergoing angiogenic process [14, 15]. The expression of this biochemical marker was decreased in cells treated with chebulagic acid compared to control. This indicates that chebulagic acid delays or inhibits angiogenesis. Another key factor involved in modulating angiogenesis is vascular endothelial growth factor. It controls a variety of endothelial cell functions involved in angiogenesis and protects endothelial cells from apoptosis [16]. VEGF may exert autocrine effect on endothelial cell inducing angiogenic phenotype. Our results showed a decreased production of VEGF in cells treated with chebulagic acid compared to control. The reduction in the production of VEGF may contribute to the antiangiogenic effect of CA. Eicosanoids particularly PGE1, are reported to cause proangiogenic effect on these model systems [17]. LOX metabolites, particularly 15-(S)-HETE, are also reported to produce proangiogenic effect on these model systems [18]. The antiangiogenic effect of CA observed here may involve inhibition of the production of such proangiogenic eicosanoids that in turn can reduce VEGF production and VEGF response. 

Pericellular proteolysis is critical during the process of angiogenesis. One of the molecular mechanisms involved in the pericellular proteolysis is the action of MMPs. Our previous results suggested the downregulation of MMPs as HUVECs undergo angiogenic process. During the initial stages, there was continuous secretion of MMP 2 and MMP 9 when HUVECs remained as single cell and their levels decreased on the later stages of culture when cell-cell contact formation occurred suggesting that MMPs are produced during the initial stages of angiogenesis, before the cell-to-cell contacts are established. Investigations using chebulagic acid showed that the production of MMP 2 and MMP 9 was not downregulated even in the later stages of culture in CA treated cells, whereas in control cells, the production of MMPs were downregulated in the later stages of culture. Decreased downregulation in the production of MMPs by chebulagic acid treated cells may contribute to the decreased cell-cell contact formation and decreased angiogenesis. 

Since endothelial cell-cell contact formation is a key event involved in the process of angiogenesis, we analyzed the effect of CA on the production of VE cadherin, which has been reported to be the major endothelial cell adhesion molecule at adherence junctions [19]. Our results showed a decrease in the production of VE cadherin in cells treated with CA compared to control cells during the later stage of the culture suggesting that the antiangiogenic effect of CA may involve downregulation of VE cadherin which may result in reduced cell-cell contact formation. 

Further investigations were carried out to understand the role of chebulagic acid in the signaling pathway downstream of VE cadherin. *β* catenin plays a major role in the downstream signaling of VE cadherin. In order to evaluate the effect of CA on the distribution of *β* catenin, nuclear and cytosolic fractions were analysed, and our results showed that about 85% of *β* catenin remained in the nucleus in both control and cells treated with CA during the initial stages of culture. But during the later stages, when the cell-cell contacts were established in control cells, *β* catenin was more localized in the cytosol. But in cells treated with CA, this translocation was less suggesting that CA inhibited the translocation of *β* catenin to the cytosol. From these results, it can be inferred that the antiangiogenic effect of CA in serum supplemented condition involved its effect on the VE cadherin *β* catenin signaling.

## 5. Conclusion

The results from our studies suggest that CA can inhibit angiogenesis in rat aortic rings and human umbilical vein endothelial cells in serum supplemented condition. CA, being a COX-LOX dual inhibitor, causes antiangiogenic effect by decreased production of the angiogenic factor VEGF. It is associated with the decrease in the production of the adhesion molecule VE cadherin and increased rate of production of MMPs which may result in increased pericellular proteolysis, decreasing VE cadherin-*β* catenin signaling.
